# Supplementary material for: Association between oral microbiome and seven types of cancers in East Asian population: a two-sample Mendelian randomization analysis
Source: Front Mol Biosci. 2023 Nov 21;10:1327893. doi: 10.3389/fmolb.2023.1327893 (PMC10702768; doi:10.3389/fmolb.2023.1327893)
Supplement: Supplementary file 1 [file DataSheet1.ZIP › Supplementary Materials/MR plots for tongue/tongue═╝/Pancreatic cancer/╥╚╧┘░⌐_╔╕╤í╜ß╣√/pheno.1939_to_pancreatic cancer_forest.pdf]

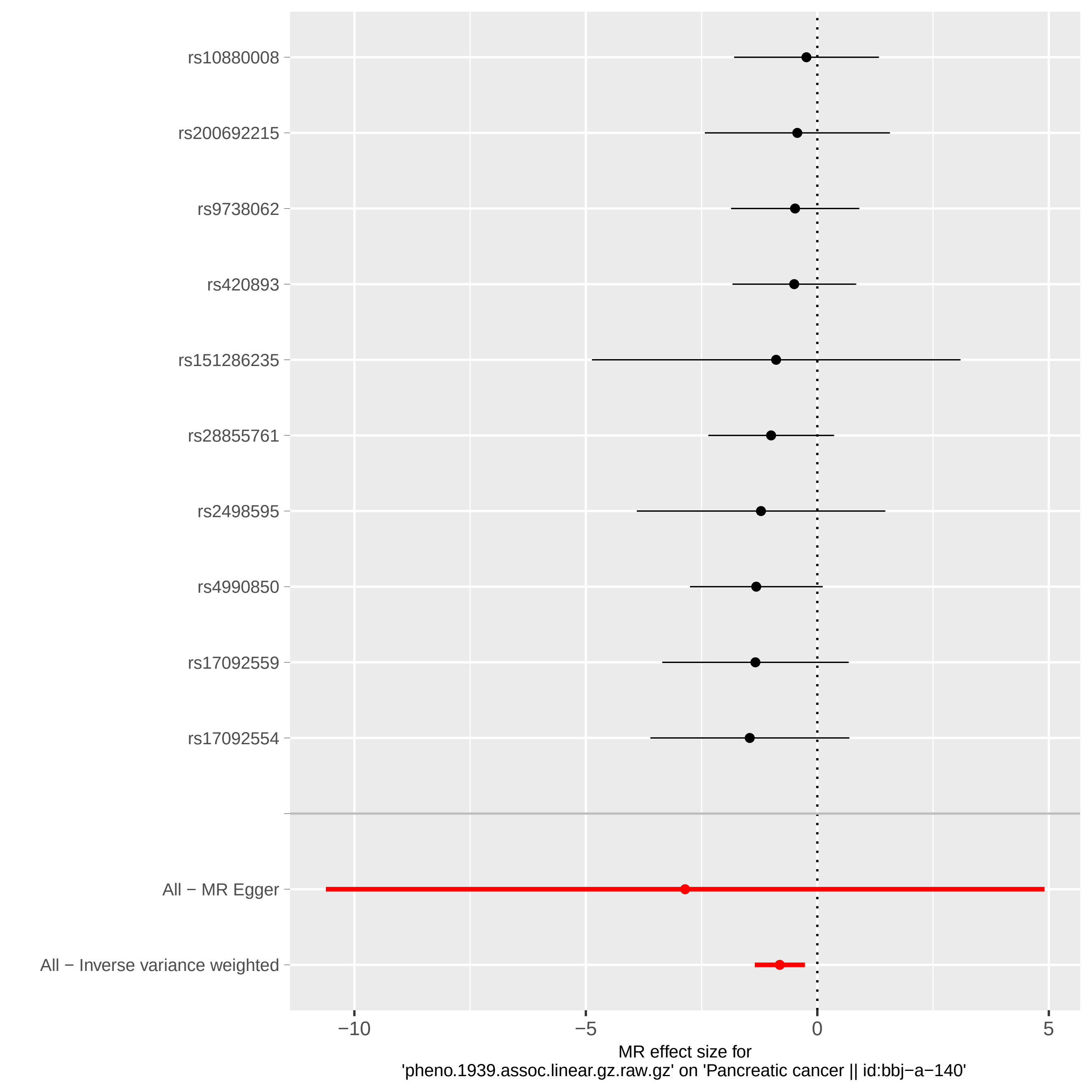

rs10880008
rs200692215
rs9738062
rs420893
rs151286235
rs28855761
rs2498595
rs4990850
rs17092559
rs17092554
All − MR Egger
All − Inverse variance weighted
−10
−5
0
5
MR effect size for
'pheno.1939.assoc.linear.gz.raw.gz' on 'Pancreatic cancer || id:bbj−a−140'
